# Impacts of discriminated PM_2.5_ on global under-five and maternal mortality

**DOI:** 10.1038/s41598-020-74437-7

**Published:** 2020-10-19

**Authors:** Patrick Opiyo Owili, Tang-Huang Lin, Miriam Adoyo Muga, Wei-Hung Lien

**Affiliations:** 1 Department of Public Health, School of Health Sciences, University of Eastern Africa, Baraton, Eldoret, Kenya; 2 Center for Space and Remote Sensing Research, National Central University, Taoyuan City, Taiwan; 3 Department of Human Nutrition and Dietetics, School of Medicine and Health Sciences, Kabarak University, Kabarak, Kenya

**Keywords:** Environmental sciences, Risk factors

## Abstract

Globally, it was estimated that maternal and under-five deaths were high in low-income countries than that of high-income countries. Most studies, however, have focused only on the clinical causes of maternal and under-five deaths, and yet there could be other factors such as ambient particulate matter (PM). The current global estimates indicate that exposure to ambient PM_2.5_ (with ≤ 2.5 microns aerodynamic diameter) has caused about 7 million deaths and over 100 million disability-adjusted life-years. There are also several health risks that have been linked PM_2.5_, including mortality, both regionally and globally; however, PM_2.5_ is a mixture of many compounds from various sources. Globally, there is little evidence of the health effects of various types of PM_2.5,_ which may uniquely contribute to the global burden of disease. Currently, only two studies had estimated the effects of discriminated ambient PM_2.5_, that is, anthropogenic, biomass and dust, on under-five and maternal mortality using satellite measurements, and this study found a positive association in Africa and Asia. However, the study area was conducted in only one region and may not reflect the spatial variations throughout the world. Therefore, in this study, we discriminated different ambient PM_2.5_ and estimated the effects on a global scale. Using the generalized linear mixed-effects model (GLMM) with a random-effects model, we found that biomass PM_2.5_ was associated with an 8.9% (95% confidence interval [CI] 4.1–13.9%) increased risk of under-five deaths, while dust PM_2.5_ was marginally associated with 9.5% of under-five deaths. Nevertheless, our study found no association between PM_2.5_ type and global maternal deaths. This result may be because the majority of maternal deaths could be associated with preventable deaths that would require clinical interventions. Identification of the mortality-related types of ambient PM_2.5_ can enable the development of a focused intervention strategy of placing appropriate preventive measures for reducing the generation of source-specific PM_2.5_ and subsequently diminishing PM_2.5_-related mortality.

## Introduction

In 2017, it was estimated that the daily maternal deaths (i.e., during pregnancy and childbirth) were over 800, with most of these deaths occurring in low- and middle-income countries (LMICs). The global variation in the maternal mortality ratio between high-income (11 deaths per 100,000 live births) and low-income countries (462 deaths per 100,000 live births) was noticeable, and this highlights the differences between the rich and the poor countries in terms of health outcomes. Moreover, the lifetime risk of maternal death was equally high in low-income countries (1 death in 45 women) than in high-income countries (1 death in 5400 women)^1^. These high maternal death rates in LMICs was also reflected in the deaths of the under-five children, which is estimated to be high in some regions like the Sub-Saharan Africa (SSA, 76 deaths per 1000 live births) as compared to that of other regions like the European region (9 deaths per 1000 live births) in 2018^2^. Nevertheless, most studies on the maternal and under-five deaths are mainly clinically-focused, and yet these deaths might not only be a result of clinical factors such as postpartum hemorrhage and eclampsia. Other factors such as environmental causes like ambient particulate matter (PM) could be related.

The current global estimates indicate that exposure to ambient PM_2.5_ (with ≤ 2.5 microns aerodynamic diameter) has caused about 7 million deaths and over 100 million disability-adjusted life-years^3^. There are also several health risks that have been linked to PM_2.5_, including mortality, both regionally and globally^3–6^; however, PM_2.5_ is a mixture of many compounds from various sources. Globally, there is little evidence of the health effects of discriminated PM_2.5_ (i.e., the major component of PM_2.5_)_,_ which may uniquely contribute to the global burden of disease. Currently, there are limited studies that have estimated the effects of discriminated or categorized ambient PM_2.5_, that is, anthropogenic, biomass and dust, on under-five and maternal mortality using satellite measurements, and these studies found a positive association in Africa^5^ and Asia^7^. However, these studies^5,7^ were regional and may not reflect the spatial variations throughout the world. Therefore, in this study, we discriminated ambient PM_2.5_ and estimated the effects on a global scale. Using the generalized linear mixed-effects model (GLMM) with a random-effects model, we found that biomass PM_2.5_ was associated with an 8.9% (95% confidence interval [CI] 4.1–13.9%) increased risk of under-five deaths, while dust PM_2.5_ was marginally associated with 9.5% of under-five deaths. Nevertheless, our study found no association between PM_2.5_ type and global maternal deaths. This result may be because the majority of maternal deaths could be associated with preventable deaths that would require clinical interventions. Identification of the mortality-related types of ambient PM_2.5_ can enable the development of a focused intervention strategy of placing appropriate preventive measures for reducing the generation of source-specific PM_2.5_ and subsequently diminishing PM_2.5_-related mortality.

Studies have indicated that sources of PM_2.5_ may vary and are likely to contribute to the accumulation of various toxic compounds that are suspended in the air, such as sulphur oxides (SO_x_), carbon monoxide (CO), particulates, and nitrogen oxides (NO_x_)^8–10^, which may then contribute to various health problems and subsequently an increase in the global burden of disease. Policy makers have also set global no-threshold limits for exposure to ambient PM_2.5_ (i.e., daily exposure less than 25 μg/m^3^ while annual exposure less than ≤ 10 μg/m^3^)^9^; yet, there is still an ongoing discussion of the need to harmonize ambient air quality standards since these standards vary greatly among regions and countries^11,12^. From these discussions, questions have also arisen as to whether each country or each region should set its own air quality standards. Nevertheless, harmonizing the national and global air quality standards may still be an issue and a challenge worthy of discussion since the point sources of ambient PM_2.5_ vary from place to place. Therefore, ambient PM_2.5_ may be linked to a variety of elements suspended in the air in different areas, which may have diverse health effects. Several authors, however, developed a modest method for identifying and quantifying different ambient PM_2.5_ types that are suspended in the air^13^, and this method has been applied to study the types of ambient PM_2.5_ and mortality in Africa^4^.

The types of ambient PM_2.5_ were measured and quantified using the same techniques, and the global effect on mortality was then estimated before a dose–response relationship in the different world regions was determined. We used the most recent satellite-based measurements of country-level annual ambient PM_2.5_ concentrations and country-level annual under-five and maternal mortality. Satellite data are important in this study because most low- and middle-income countries (LMICs) do not have adequate ground-based air quality monitoring sites that could provide real-time data. Several studies from LMICs have also used satellite data^4,5^.

Unlike one of the previous studies that focused on only the African region^4^, we employed the random-effects modelling technique using a generalized linear mixed-effects model (GLMM) with a spatial covariance structure, Poisson link function, natural cubic spline, and penalized quasi-likelihood (PQL) approach to adjust for the time, season and spatial variations in PM_2.5_ and mortality within and between different countries and regions (“Methods”). Natural spline was used as a smoothing function. The fixed-effects model is only appropriate when there is no variation between different regions or areas. However, in the case of handling global data, we expect variations between and within countries and regions; hence use of the random-effects model would provide true estimates in our analyses. The country and regional boundaries were determined before the annual means of the different types of PM_2.5_ and mortality were estimated for each country and each region (Methods; Table 1). The data over 16 years (i.e., 2000–2015) were analysed to determine the effects of the types of ambient PM_2.5_ on mortality after adjusting for potential confounders.

**Table 1:** Descriptive statistics of under-five mortality, maternal mortality, and ambient PM_2.5_ types by region.

	Total countries and islands	Under-5 mortality annual mean	Maternal mortality annual mean	Biomass PM_2.5; Jan–Dec_, μg/m^3^	Anthropogenic PM_2.5; Jan–Dec_, μg/m^3^	Dust PM_2.5; Jan–Dec_, μg/m^3^
	*n* = 206	*n* (*SD*)	*n* (*SD*)	Mean*, n* (*SD*)	Mean (*SD*)	Mean (*SD*)
**Global regions**						
**Africa**						
Central Africa	8	61,321 (104,706)	4131 (6862)	48.2 (11.3)	36.2 (8.3)	36.3 (8.2)
Eastern Africa	13	68,806 (81,033)	4613 (5546)	32.7 (5.9)	27.4 (3.6)	26.8 (4.2)
Northern Africa	6	22,022 (21,939)	706 (473)	27.3 (3.3)	24.9 (3.6)	26.7 (3.9)
Southern Africa	10	51,982 (53,012)	2310 (2335)	29.2 (3.7)	23.5 (3.4)	21.5 (2.9)
Western Africa	16	97,942 (197,426)	6061 (13,375)	41.8 (7.7)	34.9 (4.8)	35.2 (5.1)
**Americas**						
Caribbean Islands	19	12,401 (17,562)	579 (817)	34.1 (1.6)	27.7 (1.5)	29.1 (1.3)
Mesoamerica (Central)	8	9309 (14,544)	302 (404)	36.7 (4.0)	27.3 (2.2)	28.7 (3.9)
North America	3	24,185 (16,365)	821 (786)	32.1 (4.0)	29.5 (8.3)	41.1 (16.7)
South America	12	12,854 (19,641)	461 (541)	33.1 (4.9)	25.4 (3.8)	24.7 (5.8)
**Asia**						
East Asia	5	73,792 (149,993)	1446 (2709)	37.8 (6.1)	32.5 (5.5)	34.5 (6.7)
North Asia (Russia)	1	23,959 (5463)	571 (107)	30.9 (1.8)	26.7 (2.5)	42.1 (14.8)
Central Asia	5	12,458 (9356)	118 (66)	33.2 (3.3)	28.6 (3.1)	30.7 (3.3)
West Asia	16	11,225 (16,228)	340 (789)	34.2 (6.3)	29.1 (4.5)	29.9 (4.8)
South Asia	8	330,970 (605,292)	12,618 (23,035)	38.5 (6.3)	33.5 (5.9)	36.5 (7.7)
Southeast Asia	11	38,593 (52,828)	1622 (2672)	41.5 (6.6)	30.9 (3.9)	31.9 (6.1)
**Europe**						
Eastern Europe	12	2008 (2358)	30 (36)	31.8 (2.0)	26.1 (2.1)	29.2 (2.9)
Northern Europe	13	6925 (14,182)	303 (674)	31.8 (3.0)	26.2 (5.1)	29.9 (7.1)
Southern Europe	6	1551 (1298)	23 (26)	30.8 (3.4)	24.5 (2.7)	25.9 (3.2)
Western Europe	16	2905 (9610)	131 (452)	32.9 (2.8)	27.7 (2.7)	28.8 (3.2)
**Oceania**						
Australia and New Zealand	2	901 (529)	14 (6)	24.3 (0.7)	19.9 (0.6)	21.8 (1.5)
Melanesia	5	10,957 (15,496)	490 (728)	30.8 (3.6)	24.4 (2.8)	25.2 (2.7)
Micronesia	6	13,386 (18,901)	644 (878)	34.8 (0.0)	28.3 (0.0)	29.6 (0.0)
Polynesia	5	16,022 (19,682)	763 (917)	34.7 (0.0)	28.2 (0.0)	29.5 (0.0)

*n* Number, *SD* Standard deviation.

## Results and discussions

**Figure 1:**
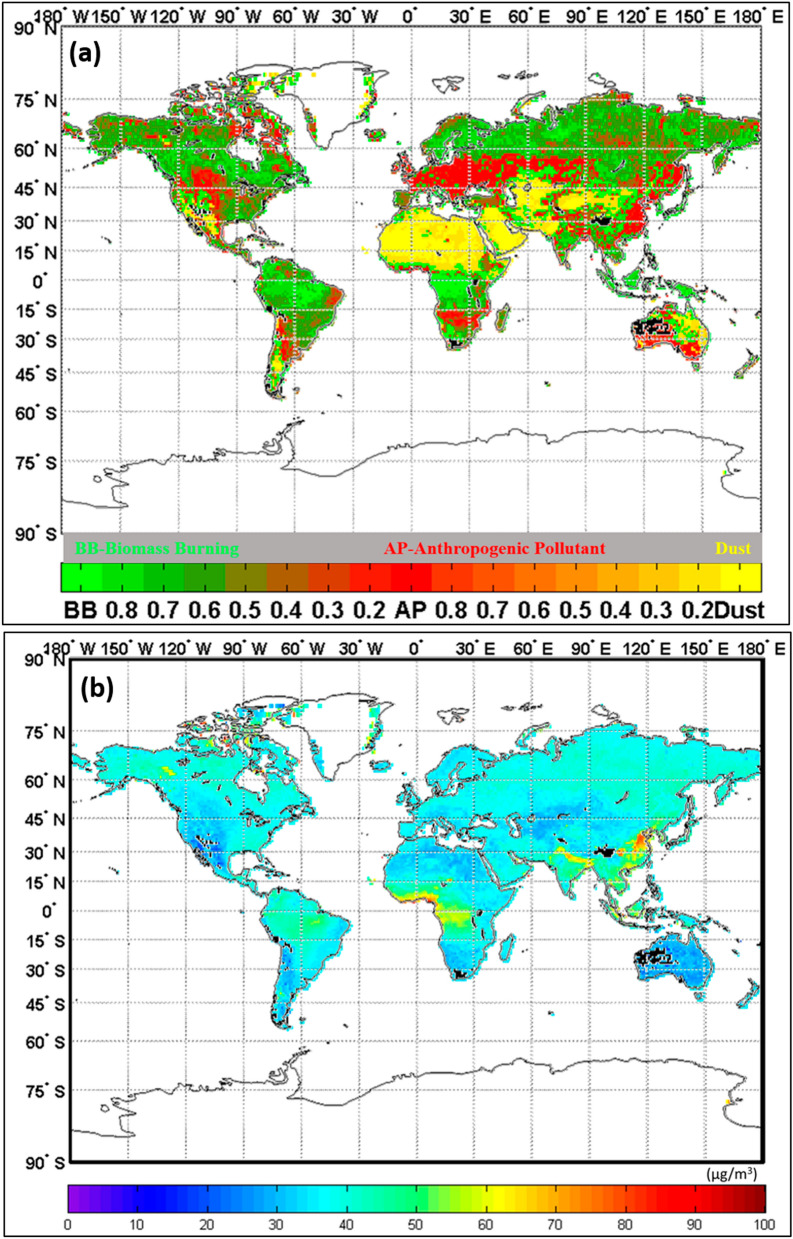
Spatial patterns of frequency and concentration of different PM_2.5_ types globally for 2000–2015. (**a**) Long-term frequency of PM_2.5_ types with fractions. (**b**) Long-term average PM_2.5_ concentration (μg/m^3^) for 2000–2015. The regions of colour in black indicate data absent. (The maps are produced by probability density function of MATLAB (matrix laboratory) software package, version 7.0, https://www.mathworks.com/products/matlab.html).

The global frequency distributions of major PM_2.5_ types (Fig. 1a) were significantly different among regions, in particular heavy PM_2.5_ loadings occurred in North America, Central Africa, West Asia, South Asia and East Asia (Fig. 1b). The impacts on human health after long-term exposure could be distinct in each region and should be carefully considered.

**Figure 2:**
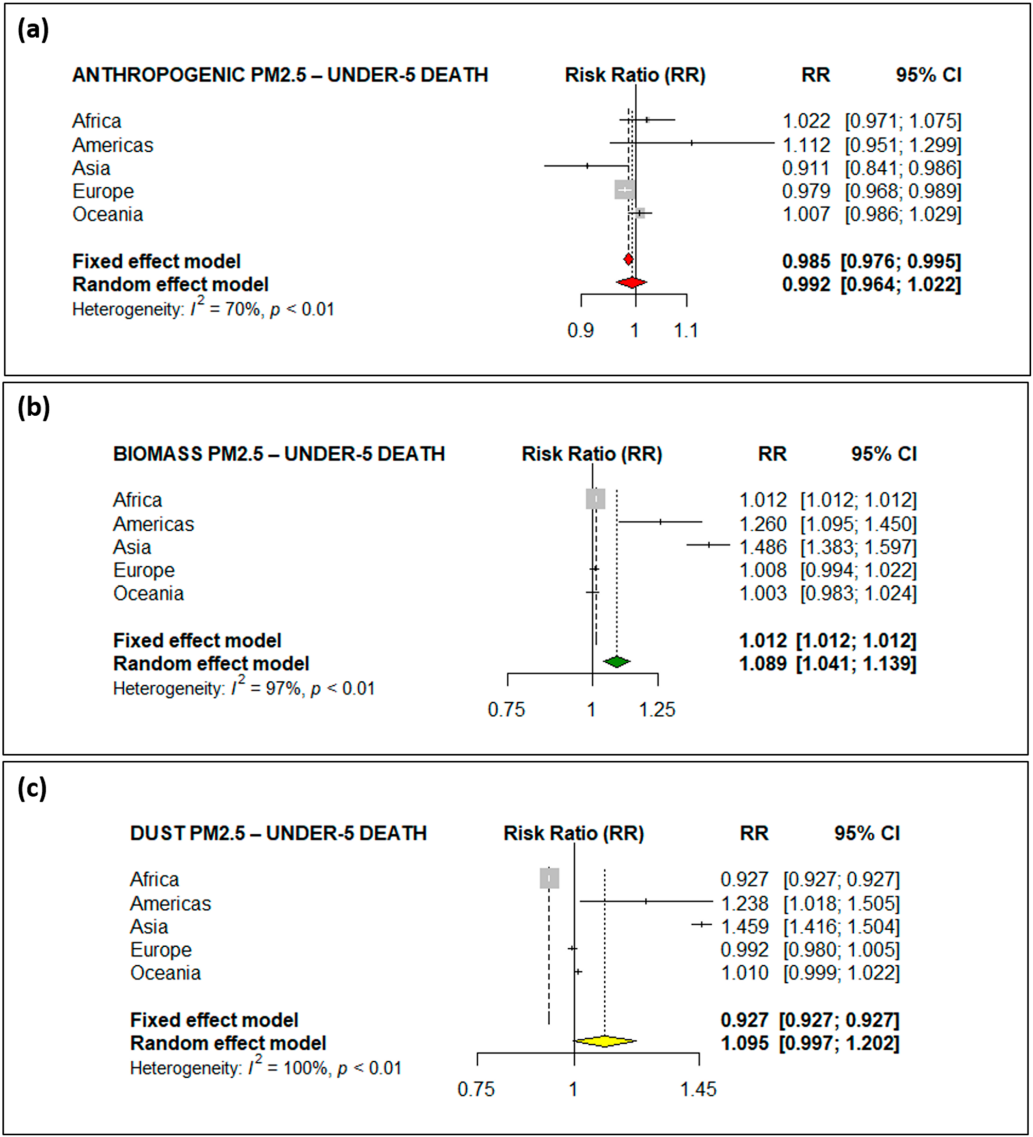
Forest plot of the risk of under-five deaths from (**a**) anthropogenic PM_2.5_, (**b**) biomass PM_2.5_ and (**c**) dust PM_2.5_ throughout the world.

**Figure 3:**
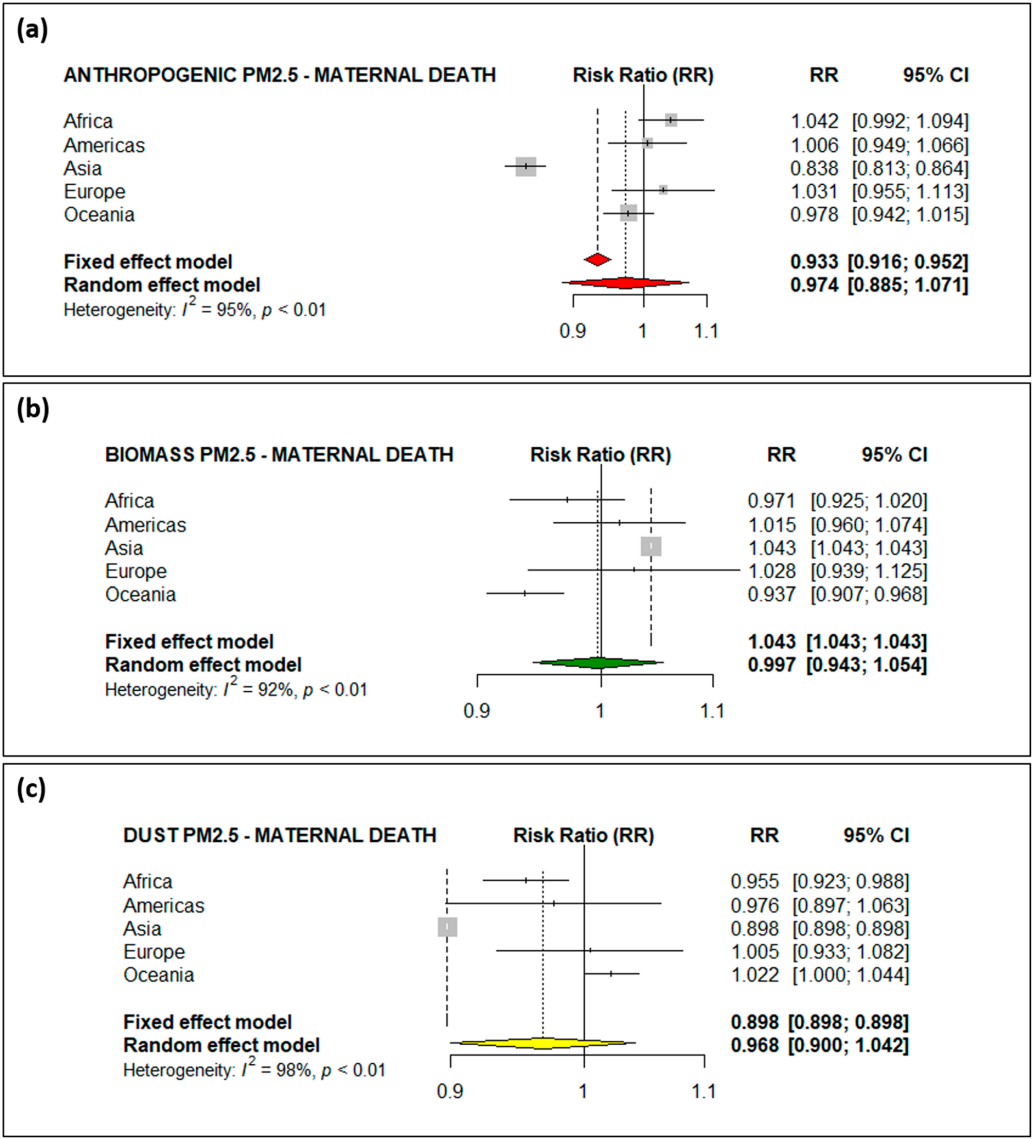
Forest plot of the risk of maternal deaths from (**a**) anthropogenic PM_2.5_, (**b**) biomass PM_2.5_ and (**c**) dust PM_2.5_ throughout the world.

A meta-analysis approach was used to estimate the global effects of the discriminated types of PM_2.5_ on under-five deaths (Fig. 2 and Table 2) and maternal deaths (Fig. 3 and Table 2). The results of the random-effects model for the global estimates indicated that biomass PM_2.5_ (Fig. 2b) was associated with an 8.9% (95% confidence interval [CI] 4.1–13.9%; *p* < 0.001) risk of under-five deaths. This result is consistent with recent evidence on non-discriminated ambient PM_2.5_, which found a 9.2% increase in infant mortality^4^. Consequently, it could possibly be argued that biomass PM_2.5_ could have contributed to a great portion of the 9.2% increase in infant mortality in the Heft-Neal, et al.^4^ study. Further testing would be necessary to show this point. The annual average biomass PM_2.5_ levels were, however, greater than 30 μg/m^3^ in most of the regions (Table 1), but the risk of death increased only in Africa by 1.2%, and the Americas and Asia contributed to the largest proportions at 26.0% and 48.6%, respectively (Fig. 2b and Table 2). The environmental health literature also suggests an association between biomass burning, which is used for cooking, and under-five mortality in different regions^14,15^.

**Table 2:** Adjusted incidence rate ratios of under-five deaths and maternal deaths.

Variable	Adjusted incidence rate ratio (95% CI)^a,b^	
	Under-5 mortality	Maternal mortality
**Anthropogenic PM**_**2.5**_		
Africa	1.022 (0.971, 1.075)	1.042 (0.992, 1.094)
Americas	1.112 (0.952, 1.299)	1.006 (0.949, 1.066)
Asia	0.911 (0.841, 0.986)*	0.838 (0.813, 0.864)***
Europe	0.979 (0.968, 0.989)***	0.992 (0.980, 1.004)
Oceania	1.007 (0.986, 1.029)	0.998 (0.942, 1.015)
**Biomass PM**_**2.5**_		
Africa	1.012 (1.012, 1.012)***	0.971 (0.925, 1.020)
Americas	1.260 (1.095, 1.450)***	1.015 (0.960, 1.074)
Asia	1.486 (1.383, 1.597)***	1.043 (1.043, 1.043)***
Europe	1.008 (0.994, 1.022)	1.028 (0.939, 1.125)
Oceania	1.003 (0.983, 1.024)	0.937 (0.907, 0.968)***
**Dust PM**_**2.5**_		
Africa	0.928 (0.928, 0.928)***	0.955 (0.923, 0.988)**
Americas	1.238 (1.018, 1.506)**	0.976 (0.887, 1.063)
Asia	1.460 (1.417, 1.506)***	0.898 (0.898, 0.898)***
Europe	0.992 (0.980, 1.005)	1.005 (0.933, 1.082)
Oceania	1.010 (0.999, 1.022)	1.022 (1.000, 1.044)

*CI* confidence interval.

*p ≤ 0.05; **p ≤ 0.01; ***p ≤ 0.001.

^a^In one unit increments of PM_2.5_ concentration.

^b^Generalized linear mixed-effects models (GLMM) random-effect is used with natural cubit spline for smoothing.

The dust PM_2.5_ (Fig. 2c and Table 2) marginally increased the risk of under-five mortality by 9.5% (*p* = 0.058). However, the increased risk of death was statistically significant in only the Americas and Asia at 23.8% and 45.9%, respectively. Saharan dust events (i.e., African dust storms) inject large amounts of mineral dust into the air over the Atlantic Ocean and have been linked to the increase in PM_2.5_ in North America, Central America, the Caribbean and Europe in the months between June and October^16–19^. Our study also found that the annual mean levels of dust PM_2.5_ were relatively high in North America and Asia compared to other regions (Table 2). Ironically, the desert area of Northern Africa had low levels of annual mean dust PM_2.5_ (26.7 μg/m^3^), possibly because larger particles settle very fast in the area, while small particles (PM_2.5_) remain suspended in the air and are then transported to other regions by wind^19^. Dust storms are natural phenomena that are not associated with local economic activity, and the effects can be reduced only when appropriate health and safety measures and environmental control strategies are considered, such as using dust masks, increasing the vegetation cover, and designing buildings appropriately.

We also found no relationship between anthropogenic PM_2.5_ and under-five mortality using the random-effects model (Fig. 2a). There was only an 11.2% increase in the risk of under-five deaths in the Americas region, but this increase was not statistically significant. This finding may be possibly explained by the assumption that under-five children spend much of their time indoors, unlike adults. Notwithstanding this reason, other possible confounders and limitations of actual exposure measurements may help in explaining the results.

The global estimates, however, indicated a lack of association between different types of ambient PM_2.5_ and maternal deaths (Fig. 3), except for a positive relationship between biomass PM_2.5_ and maternal deaths in Asia (Fig. 3b), with a 4.3% increased risk of death. Most mothers from low-income households use biomass fuel for their daily cooking and thus have an increased risk of exposure to biomass PM_2.5_^20–22^. It is therefore imperative to think that most maternal deaths are clinically related, which would then require clinical solutions to maternal deaths. It is still important to adequately estimate how much of these deaths are contributed by PM_2.5_.

**Figure 4:**
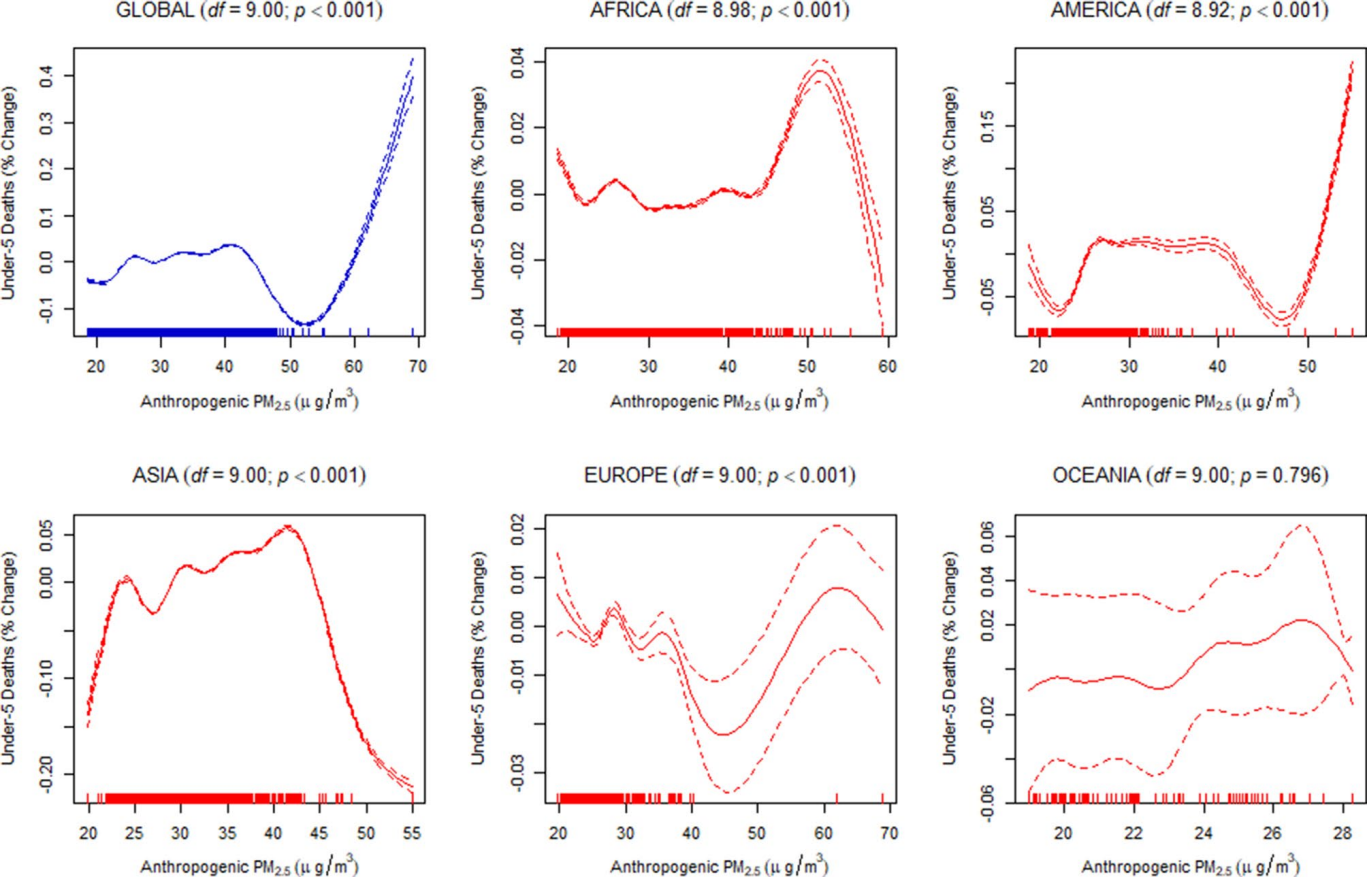
Penalized spline of anthropogenic PM_2.5_ and under-five deaths by region.

**Figure 5:**
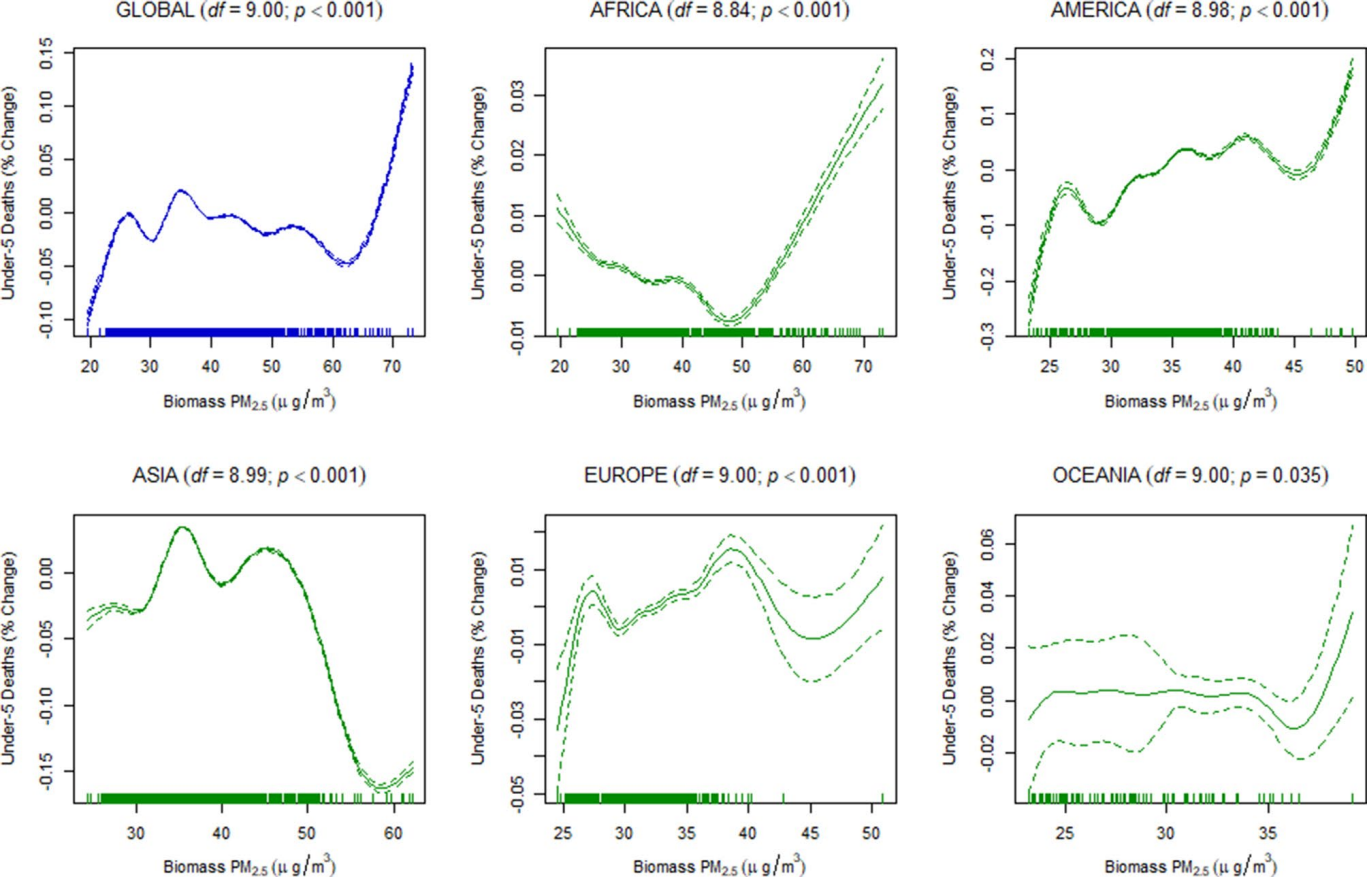
Penalized spline of biomass PM_2.5_ and under-five deaths by region.

**Figure 6:**
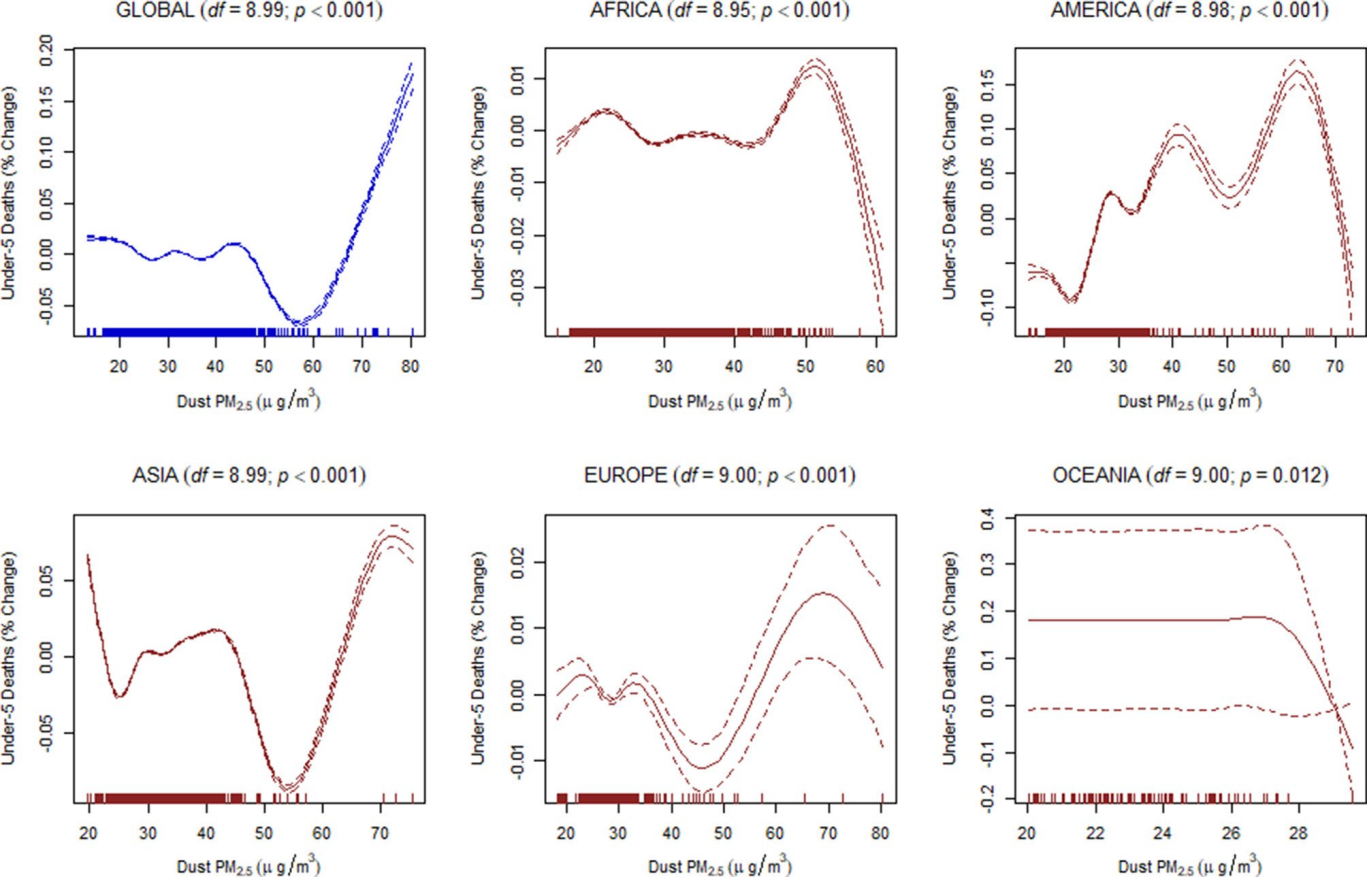
Penalized spline of dust PM_2.5_ and under-five deaths by region.

**Figure 7:**
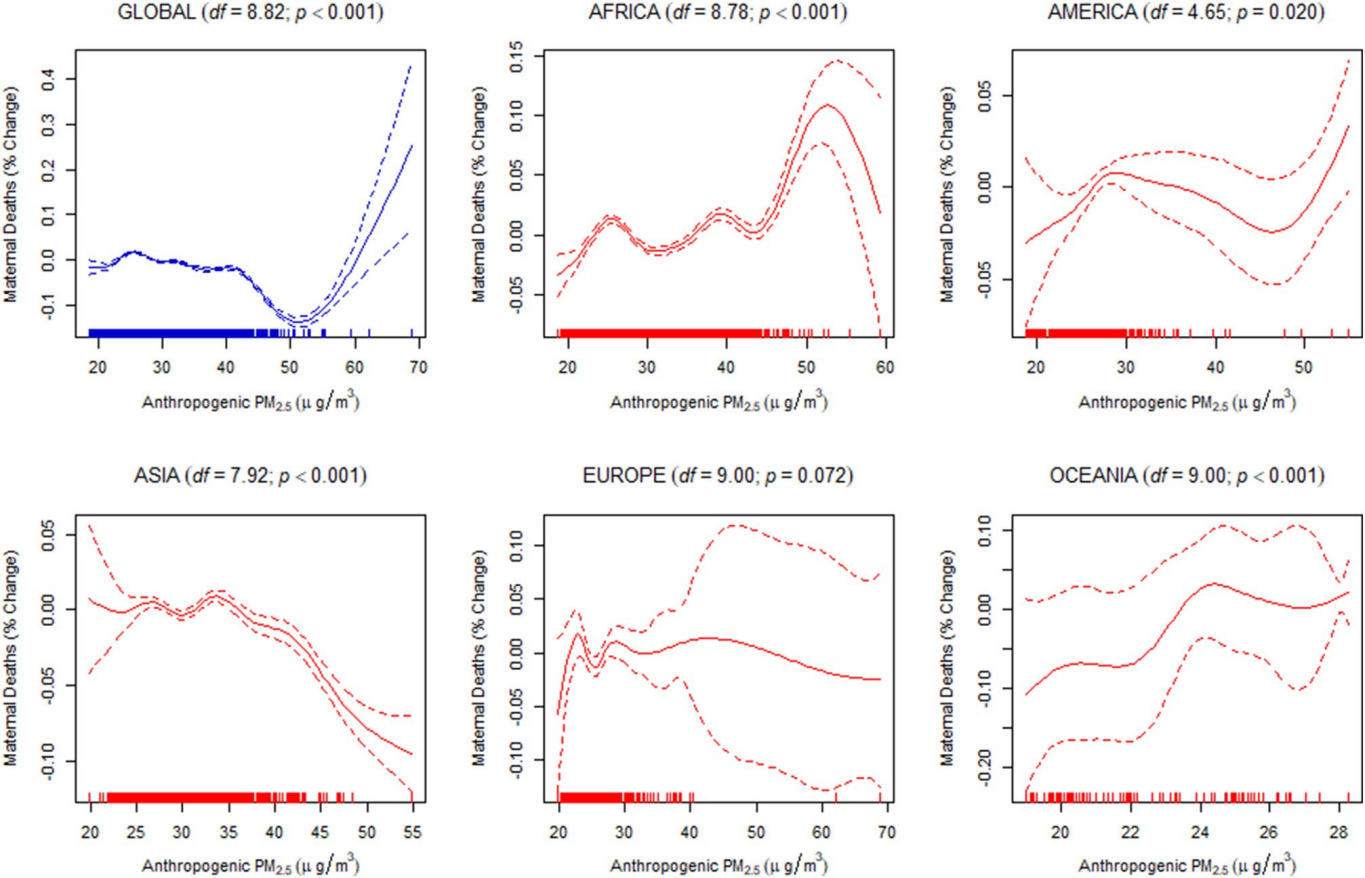
Penalized spline of anthropogenic PM_2.5_ and maternal deaths by region.

**Figure 8:**
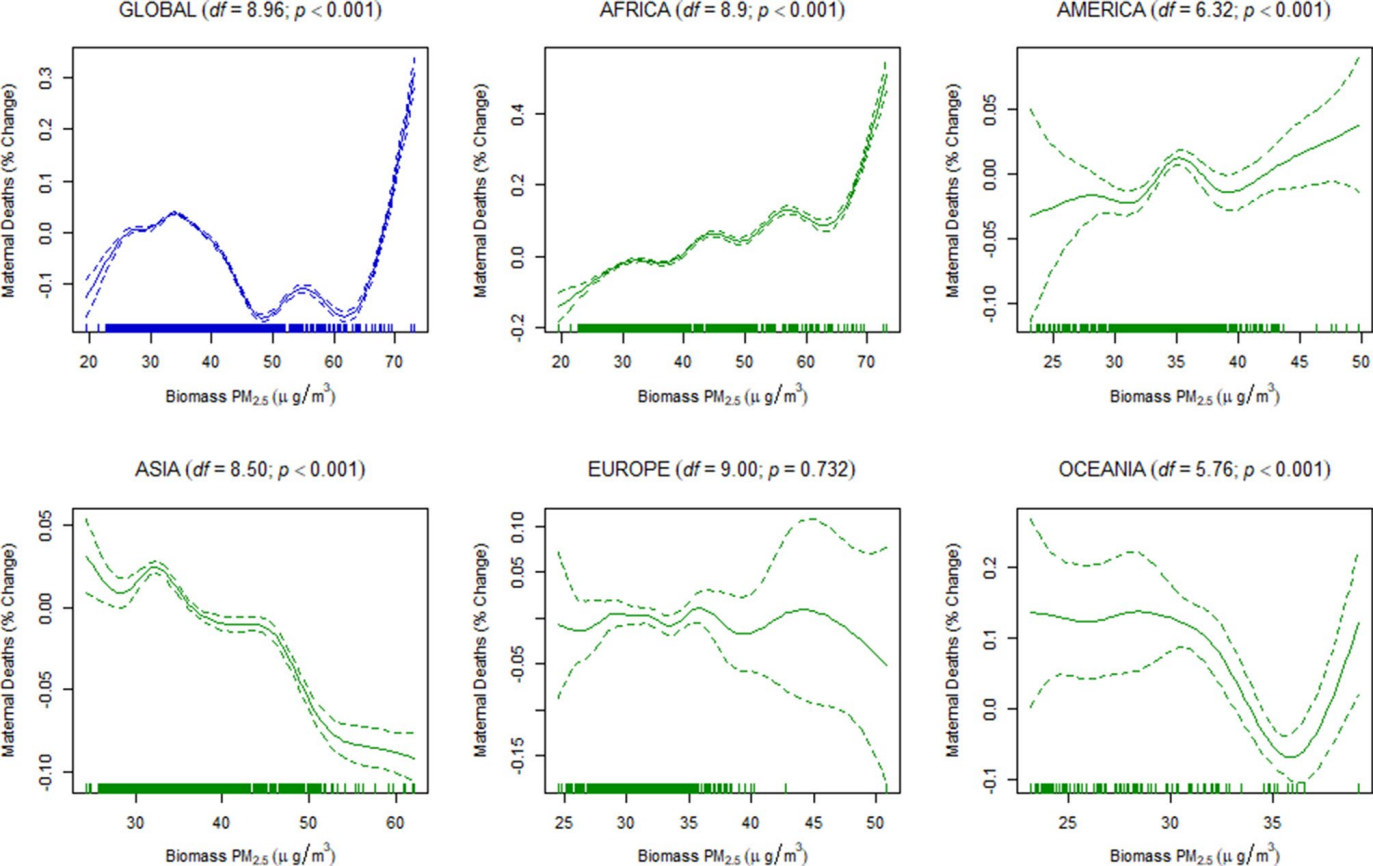
Penalized spline of biomass PM_2.5_ and maternal deaths by region.

**Figure 9:**
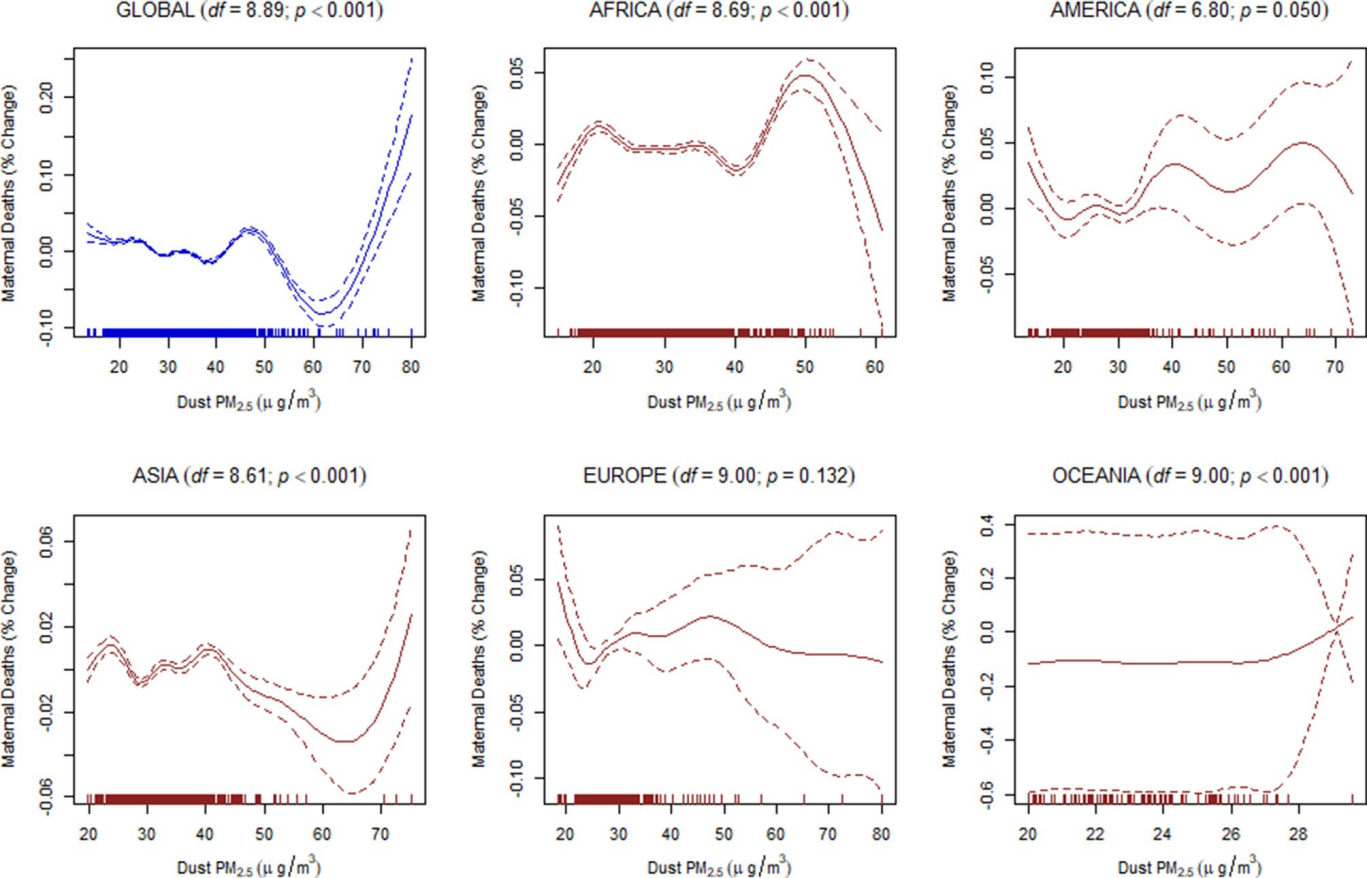
Penalized spline of dust PM_2.5_ and maternal deaths by region.

Finally, to estimate the dose–response relationship between the discriminated ambient PM_2.5_ and the under-five and maternal mortality, we used a generalized additive mixed-effects model (GAMM) with a random-effect estimation procedure (Methods). Since estimations of the dose–response relationship for global data may not be linear, the penalized spline smoothing function was used to determine the non-linear relationship between the discriminated ambient PM_2.5_ and the under-five and maternal mortality (Figs. 4, 5, 6, 7, 8, 9). However, the results on the dose–response relationship of the global biomass PM_2.5_ and the under-five mortality and maternal mortality indicated a slight increase in the risk of under-five deaths (Fig. 8) and maternal deaths (Fig. 8) after surpassing a biomass PM_2.5_ concentration of approximately 33 μg/m^3^, suggesting higher levels of exposure than the current global standards, which require daily exposure to be less than 25 μg/m^3^ while annual exposure should be less than ≤ 10 μg/m^3^. In our analyses, however, we were unable to determine the global no-threshold levels for air quality standards because of the nonlinearity of the data. Consequently, discussions of global standards and further research are still necessary if revisions are to be made. Moreover, the populations in different regions might also have developed a stronger immune response and resilience to several hazardous elements, thereby increasing the no-threshold limit; this hypothesis warrants more tests and discussions.

Our study has several limitations and strengths. One major limitation is in our study design, which is linked to ecological fallacy—this limits our findings to an aggregate, which cannot be deduced or inferred to an individual. Future studies focused on several high-risk populations and individuals are important to extend the findings of this study. Secondly, we were unable the to classify the PM_2.5_-specific mortality, and the use of all-cause mortality is a major limitation in the outcome indicator. Some of the deaths in different countries might have been linked to either clinical or non-clinical factors that are not environmentally-related. This may limit causality. It is therefore necessary that future studies be aligned to cause-specific mortality which can then generate the ambient PM_2.5_-specific death point estimates. Thirdly, there are other individual and environment factors that we were unable to control for in this study such as demographic characteristics of individuals, precipitation and humidity. These potential confounders were not controlled for because of data limitations. Several studies that would identify and collect several potential confounders are very important. Lastly, the individual level of exposure to PM_2.5_ cannot be adequately determined in this study, and hence it is one of the limitation towards obtaining the true dose–response relationship. There is need of an accurate exposure assessment in a follow-up study so that an accurate dose–response relationship can be determined. Our result should, therefore, be interpreted with a lot of caution since this study only determined a temporal causality.

However, our study had several strengths. First, this is the first study to investigate the link between ambient PM_2.5_ and the global maternal and under-five deaths. Second, the analytical strategy used in this study is very comprehensive. Advanced parametric and nonparametric analytical techniques were used to analyze the data. Estimation of dose–response relationship associated with ambient PM_2.5_ exposures is a major challenge for researchers because of its nonlinearity^23^. Our study used these two approaches to assess the effect of ambient PM_2.5_ in a linear and nonlinear approaches. Finally, the spatial domain in this study covered the entire world wide which is important towards understanding the global effect of ambient PM_2.5_.

## Conclusions

Our study estimated the effects of discriminated ambient PM_2.5_ on the death of the under-five children and their mothers, using the global data of all the countries from different regions of the world, unlike others^3–5^. The PM_2.5_ from biomass was associated with the global under-five mortality. Close to two decades of satellite and mortality measurements in each country made it possible to estimate the effect of chronic exposure to various types of ambient PM_2.5_ on Earth. Our results suggest that poor air quality is a contributor to global under-five deaths and that appropriate air monitoring techniques and intervention strategies should be put in place to reduce the global burden of disease. It is also of great importance to determine the actual deaths that are attributable to PM_2.5_ to enable a conclusive determination of the no-threshold limits for different regions and, subsequently, the global air quality standards.

With the new development of remote sensing technology for aerosol portioning, the global impacts of discriminated PM_2.5_ on under-five and maternal mortality are carefully examined for the first time using satellite observations. The results illustrated that the different sensitivity of under-five and maternal mortality to the types of PM_2.5_ in regional shown in Figs. 2 and 3 and highly correspondent with the PM_2.5_ concentration as Fig. 1b demonstrated in the regions of Asia and Africa. This research represents a significant advance in public health science related to species of air pollution in daily life, which is currently one of the greatest global issues.

## Methods

### Spatial domain

The spatial domain included 206 countries and islands that were listed according to the ISO 3166 list of countries maintained by the International Organization of Standardization (Table 3). These countries were grouped according to different geographical locations for our analyses.

**Table 3:** List of countries following the ISO 3166 list of countries and islands maintained by the International Organization of Standardization, assigned to each geographical region.

Countries and islands by regions and sub-regions
**Africa**
**Central Africa**
Burundi, Central African Republic, Chad, Congo, Congo (The DRC), Equatorial Guinea, Gabon, and Sao Tomé and Principe
**Eastern Africa**
Comoros, Djibouti, Eritrea, Ethiopia, Kenya, Madagascar, Mauritius, Rwanda, Seychelles, Somalia, Sudan (including South-Sudan), Tanzania, and Uganda
**North Africa**
Algeria, Egypt, Libya, Mauritania, Morocco, and Tunisia
**Southern Africa**
Angola, Botswana, Kingdom of Eswatini (formerly Swaziland), Lesotho, Malawi, Mozambique, Namibia, South Africa, Zambia, and Zimbabwe
**West Africa**
Benin, Burkina Faso, Cameroon, Cape Verde, Côte d'Ivoire, Gambia, Ghana, Guinea, Guinea-Bissau, Liberia, Mali, Niger, Nigeria, Senegal, Sierra Leone, and Togo
**Americas**
**Caribbean Islands**
Anguilla, Antigua and Barbuda, Aruba, Bahamas, Barbados, Bermuda, Bonaire (St Eustatius & Saba), Cayman Islands, Cuba, Curaçao, Dominica, Dominican Republic, Grenada, Guadeloupe, Haiti, Jamaica, Martinique, Montserrat, Puerto Rico, Saint Bathélemy, Saint Kitts and Nevis, Saint Lucia, Saint Martin (French Part), St Vincent & the Grenadines, Sint Maarten (Dutch Part), Trinidad and Tobago, Turks and Caicos Islands, Virgin Islands (British), and Virgin Islands (U.S.)
**Mesoamerica (Central)**
Belize, Costa Rica, El Salvador, Guatemala, Honduras, Mexico, Nicaragua, and Panama
**North America**
Canada, Greenland, and United States
**South America**
Argentina, Bolivia (Plurinational State of), Brazil, Chile, Colombia, Ecuador, Falkland Islands (Malvinas), French Guiana, Guyana, Paraguay, Peru, Suriname, Uruguay, and Venezuela (Bolivarian Rep)
**Asia**
**East Asia**
China, Japan, Korea (Dem People's Rep of), Korea (Republic of), and Mongolia
**North Asia**
Russian Federation
**Central Asia**
Kazakhstan, Kyrgyzstan, Tajikistan, Turkmenistan, and Uzbekistan
**West Asia**
Bahrain, Cyprus, Iran (Islamic Republic of), Iraq, Israel, Jordan, Kuwait, Lebanon, Oman, Palestine (State of), Qatar, Saudi Arabia, Syrian Arab Republic, Turkey, United Arab Emirates, Yemen, and West Bank & Gaza
**South Asia**
Afghanistan, Bangladesh, Bhutan, India, Maldives, Nepal, Pakistan, and Sri Lanka
**Southeast Asia**
Brunei Darussalam, Cambodia, Indonesia, Laos People's Dem Republic, Malaysia, Myanmar, Philippines, Singapore, Thailand, Timor-Leste, and Viet Nam
**Europe**
**Eastern Europe**
Albania, Armenia, Azerbaijan, Belarus, Bulgaria, Bosnia and Herzegovina, Georgia, Moldova, Montenegro, Romania, Serbia, and Ukraine
**Northern Europe**
Denmark, Estonia, Faroe Islands, Germany, Iceland, Ireland, Isle of Man, Latvia, Lithuania, Netherlands, Norway, Sweden, and United Kingdom
**Southern Europe**
France, Greece, Italy, Malta, Portugal, and Spain
**Western Europe**
Andorra, Austria, Belgium, Croatia, Czech Republic, Finland, Hungary, Liechtenstein, Luxembourg, Macedonia (formerly Yugoslavia), Monaco, Poland, San Marino, Slovakia, Slovenia, and Switzerland
**Oceania**
**Australia and New Zealand**
Australia and New Zealand
**Melanesia**
Fiji, Papua New Guinea, Solomon Islands, Vanuatu, and New Caledonia
**Micronesia**
Micronesia (Federated States), Guam, Kiribati, Marshall Islands, Nauru, Northern Mariana Islands, and Palau
**Polynesia**
American Samoa, French Polynesia, Samoa, Tonga, and Tuvalu

### Under-five and maternal mortality data

The mortality data used in our study were from the World Bank on the annual deaths of children who were 5 years and younger and that of mothers who died during pregnancy^24^. These were count data from 2000 to 2015. Each country reported the total number of deaths during a given year, and this became our outcome of interest.

### Discrimination of ambient PM_2.5_

The Moderate Resolution Imaging Spectroradiometer (MODIS) aerosol optical depth products (MYD04/Aqua and MOD04/Terra) were used to derive the PM_2.5_ concentrations^25^. The spectral aerosol optical depth (AOD) was then used to classify different categories of PM_2.5_, that is, anthropogenic, biomass burning and dust. The methods used to derive the spatial and temporal exposure patterns were explained in one study^26^ and applied in another^5^. The optical properties of particle size distribution and single scattering albedo (absorption and scattering) are important to distinguish between aerosol types, while the concentrations were calculated using the AOD-PM_2.5_ association for each type of aerosol^27^. The PM_2.5_ (*μ*g/m^3^) types were generated using the following formulas^27^:

$$ PM_{2.5}^{Biomass} = 98.3 \times AOD_{{660\,{\text{nm}}}} + 15.4; $$

$$ PM_{2.5}^{Anthropogenic} = 62.4 \times AOD_{{660\,{\text{nm}}}} + 12.4;\,{\text{and}} $$

$$ PM_{2.5}^{Dust} = 52.8 \times AOD_{{660{\text{nm}}}} + 9.68 . $$

### Potential confounders

The following country-level variables from 2000 to 2015 were used to adjust our model: total number of undernourished, anaemic pregnant women, tuberculosis cases, AIDS deaths, employed, females, population in urban areas, year, country and country’s annual mean temperature. All the data were extracted from the World Bank’s database^24^.

### Statistical treatment

After data cleaning, our data were analysed in several stages. First, the monthly PM_2.5_ concentrations were used to generate the annual average concentrations for each country and subsequently for each region. The annual mean mortality and types of PM_2.5_ are presented for each region (Table 1). Second, data were analysed using the penalized quasi-likelihood (PQL) approach in the generalized linear mixed-effects model (GLMM) with a spatial covariance structure and the Poisson link function to obtain the adjusted incident rate ratio (IRR) for each region (Table 2). A natural cubic spline was employed for the smoothing effect while specifying the country and year as the random effects since both the outcome and the exposure were dispersed and correlated over time and across boundaries^28^. We also considered spatial variations by employing a spatial covariance structure in our analyses. Third, a meta-analysis approach was used to determine the global estimates of the adjusted risk of death as a result of the PM_2.5_ types (Figs. 2, 3). Finally, the dose–response relationship was determined using the penalized spline and the generalized additive mixed-effects model (GAMM), and year and country were taken as the random effects because of the nonparametric relationship that was exhibited in the global data (Figs. 4, 5, 6, 7, 8, 9). The degrees of freedom were estimated using generalized cross-validation (GCV). We stratified all our analyses by different geographical regions in the world. The GAMM accounts for the over-dispersion and correlation in an additive non-linear approach, as it considers the random effects in the additive predictor. Moreover, GAMM also uses nested and crossed designs to analyse spatial, clustered and hierarchical data^28^. R version 3.6.0^29^ software and Stata version 13.0^30^ were used in our analyses. Data were then presented in the forms of tables and figures.

## Data Availability

Mortality data used for our analyses are available from the World Bank databank (databank.worldbank.org) while the PM_2.5_ data were extracted from the MODIS aerosol optical depth satellite products (https://modis.gsfc.nasa.gov/data/dataprod/mod04.php).
